# Running Energy Reserve Index (RERI) as a new model for assessment and prediction of world, elite, sub-elite, and collegiate running performances

**DOI:** 10.1038/s41598-023-29626-5

**Published:** 2023-05-07

**Authors:** Govindasamy Balasekaran, Mun Keong Loh, Peggy Boey, Yew Cheo Ng

**Affiliations:** Human Bioenergetics Laboratory, Physical Education and Sports Science, National Institute of Education, Nanyang Technological University, 1 Nanyang Walk, Singapore, 637616 Singapore

**Keywords:** Fat metabolism, Homeostasis

## Abstract

The purpose of this study was to utilize the Running Energy Reserve Index (RERI) model and two-trial procedure to predict all-out athletic performances. Twenty-nine trained athletes tested for differences between RERI_E_ and RERI_spd_ (hypothesis 1). Six sprint trained (ST), six middle distance (MD), and six endurance trained (ET) athletes were selected to test for differences in the value of the constant. The prediction of all-out run performances using the RERI model (hypothesis 2) and two treadmill trials procedure (hypothesis 3) were tested on eighteen trained athletes. Lastly, three trained athletes were utilized to predict all-out running performances utilizing two track trials equation (hypothesis 3). RERI_E_ and RERI_spd_ were significantly different between ST, MD, and ET athletes. The RERI_E_ model with a fixed c_E_ value of 0.0185 s^−1^ predicted all-out running performances to within an average of 2.39 ± 2.04% (R^2^ = 0.99, n_T_ = 252) for all athletes, with treadmill trials to within an average of 2.26 ± 1.89% (R^2^ = 0.99, n_T_ = 203) and track trials to within an average of 2.95 ± 2.51% (R^2^ = 0.99, n_T_ = 49). The two trials equations predicted all-out track performances to within errors of 2.43%. The RERI model may be accurate in determining running performances of 200 m and 5000 m, and treadmill performances ranging between 5 and 1340 s with a high level of accuracy. In addition, the two-trial procedure can be used to determine short and middle distance running performances of athletes and world-class runners.

## Introduction

In athletic running performances, various theoretical, empirical, mathematical, and physiological models have been developed to predict running performances ranging from 100 m to 42.4 km^1^. Most of these models were based on the speed-duration relation and human bioenergetics proposed by Hill in 1926 and 1950^2–4^. These models predicted sprint, middle-, and long-distance running performances using exponential^5^, hyperbolic^6^, logarithmic^7,8^, and polynomial functions^9^. However, these models may produce high errors or may not be suitable for all types of athletes. This study aimed to investigate a model that could predict any running event performance based on human bioenergetics with reduced errors.

Medbo and other researchers compared maximal accumulated oxygen deficit (MAOD) with available measures of anaerobic energy during short-term high intensity exercises. There were significant correlations between MAOD and (1) Wingate’s measures of anaerobic power (r = 0.69–0.70, *p* ≤ 0.05^10^), (2) treadmill run (r = 0.62–0.66, *p* ≤ 0.05^10^), (3) peak 5 min post-exercise blood lactate (BLa) (r = 0.44–0.82^10–12^), (4) 300 m time (r = − 0.76, *p* ≤ 0.01^10^), (5) 400 time (r = − 0.57, *p* ≥ 0.05^10^), and (6) 300 m shuttle run (r = 0.75^13^). The results indicated no significant differences between MAOD measured in the laboratory and on the track. The MAOD was significantly correlated with changes in blood and muscle metabolites during whole body exercise (r = 0.94^14^) and small muscle group exercises (r = 0.95–1.00^15^). These findings suggest a strong relationship between MAOD and other measures of anaerobic energy.

Other studies also revealed that parabolic power-velocity had high correlation that predicted power outputs (in relation to functional movement tasks) at a “two-point method” under two velocity conditions^16,17^. However, most of these models were either unable to account for the progressive reduction of maximal aerobic energy sustained beyond 3000 m^18–20^, or were based on unverified assumptions^21^.

The speed-duration relation has generally been used to determine anaerobic energy reserve^22^, with three main models being developed. The first model assessed all-out running performances of durations from 100 to 800 s^22,23^. While the model involved experimental data to estimate and measure the values for predicting performances, the prediction error was high for 800 m (13.8%) to 5000 m (2.4%). Furthermore, the application of this model was not extended to sprint performances, possibly due to the limitations in accurately measuring anaerobic energy^24–26^. The second model was based on theoretical consideration of world records, in which no direct or practical metabolic measurements of athletes were incorporated^21,27,28^. Even though this model had an error of less than 2%, the validity of this model on experimental data is unknown.

The third model was developed by Bundle et al.^5^ and is termed as anaerobic speed reserve (AnSR). AnSR is defined as the difference between maximal anaerobic speed (MAnS) and maximal aerobic speed (MAS), or the difference between maximal speed utilizing maximal anaerobic energy (Spd_an_) and maximal speed utilizing maximal aerobic energy (Spd_aer_). The use of AnSR has accurately predicted sprinting^5,29^, middle distance running^22,23^, cycling, and swimming^30^ performances.

AnSR utilizes negative exponential relation between all-out run durations and corresponding speeds. This approach has successfully predicted track (100–400 m) and treadmill (3–240 s) performances to within an average of 3.4% (*R*^*2*^ = 0.86) and 2.5% (*R*^*2*^ = 0.94) respectively. These findings validated the calculation of AnSR based on anaerobic and aerobic energy release via maximal speeds, and the prediction of run performances via anaerobic energy reserve. In addition, Bundle’s method only requires a simple equation using two all-out run trials of approximately 3 s and 60 s. This method predicted all-out track (100–400 m) and treadmill performances to within an average of 3.3% (*R*^*2*^ = 0.89, number of trials (*n*_*T*_) = 28) and of 3.7% (*R*^*2*^ = 0.93, *n*_*T*_ = 77) respectively.

While the AnSR method is easy and convenient for users to predict speed running performances, it is not validated for predicting all-out running performances beyond 240 s. The requirement of sophisticated equipment such as timing gates or photocells to determine top speed attained during 55 m sprint run test may also decrease its utility in daily routine running performance testing. Therefore, this study aimed to develop a new model termed running energy reserve index (RERI) based on Bundle’s AnSR concept to accurately predict all-out running performances even beyond 240 s. RERI has been defined as the ratio of oxygen uptake ($\dot{\text V}{\text O_2}$)﻿ at MAnS (E_MAnS_) to $\dot{\text V}{\text O_2}$ at MAS (E_MAS_), termed as RERI_E_; as well as the ratio of MAnS to MAS, termed as RERI_spd_. MAS (further explained under Methods) used in this study is a new framework that has been developed and validated in Part 1 of this study^31^.

This paper is the second part of the study that utilized a newly validated MAS framework (Part 1)^31^ to calculate RERI. The RERI was used to predict athletic performances for short to middle distance on the track and treadmill. Three hypotheses were developed for this study. (1) RERI_E_ and RERI_spd_ will be similar for all athletes (world, elite, sub-elite and collegiate). (2) There will be no difference between the value of constant among sprint trained (ST), middle-distance (MD), and endurance trained (ET) athletes in the prediction of all-out run performances using the RERI model (RERI_E_ and RERI_spd_ along with constant will accurately predict all-out run performances ranging from 5 to 1500 s approximately with 200 m and 5000 m on the track, and 5–1340 s approximately on the treadmill). (3) A two-trial equation based on RERI_E_ and RERI_spd_ will accurately predict all-out running performances of athletes and world-class performers.

## Methods

### Participants

Twenty-nine trained athletes (age: 27.8 ± 7.8 years, height: 175.4 ± 6.4 cm, BMI: 22.3 ± 1.8 kg·m^−2^, body fat percentage (BF%): 12.8 ± 3.1%) were selected to test the first hypothesis. 9 ST (N = 3), MD (N = 3), and ET (N = 3) athletes (age: 24.6 ± 7.13 years; height: 173.9 ± 8.84 cm; BMI: 21.2 ± 2.28 kg·m^−2^) were selected for hypothesis 2. Eighteen trained athletes were selected to predict the all-out running performances using the RERI model (hypothesis 2) and two treadmill trials procedure (hypothesis 3). Lastly, 3 trained athletes (age: 23.0 ± 0.0 years, height: 166.6 ± 11.3 cm; BMI: 20.1 ± 0.7 kg·m^−2^) were utilized to predict all-out running performances utilizing two track trials equation (hypothesis 3).

The trained athletes were either specialized in track events such as 100–400 m races (ST); training for middle distance events such as 800–3000 m (MD); competing or competed regularly in triathlons such as the ironman distance race (2.86 km swim, 180.25 km bike, and 42.20 km run), 5000 m, 10,000 m, half marathons, and full marathons (ET); or participating in other sports where both aerobic and anaerobic energy systems have a contribution.

All participants were informed of the risk and benefits of the study, and informed consent was obtained from the participants. This study was approved by the Physical Education ethics review committee of National Institute of Education, Nanyang Technological University, Singapore. All methods were performed in accordance with the relevant guidelines and regulations.

### Experimental design

The experimental design consisted of a within participant cross-sectional design, where each participant completed five to six exercise sessions in the laboratory and six sessions on the track. These participants were also involved in the validity of two trials equation to determine RERI and predict all-out running performances that consisted of four additional track protocols.

Participants were instructed to avoid strenuous activities, alcohol, and caffeine 24 h before testing.

To test the second hypothesis, a curve of individual estimated energy demand and all-out speeds at the corresponding running duration with a mathematical function was established, and the agreement between experimental and predicted (using RERI model) track (200 m and 5000 m) and treadmill performances was determined. Energy demand-duration relation and speed-duration relation of the athletes confirmed the rational mathematical functions as follows:

1 $$E\left( t \right) = \frac{{E_{MAnS} + b_{E} t}}{{1 + c_{E} t}}$$

2 $$Spd\left( t \right) = \frac{{MAnS + b_{spd} t}}{{1 + c_{spd} t}}$$

where, estimated energy (*E*) and speed (*Spd*) decreased with increasing corresponding running duration (*s*) in a rational function. E_MAnS_ (ml·kg^−1^·s^−1^) represents maximal anaerobic energy, while MAnS (m·s^−1^) represents maximal anaerobic speed. *b*_*spd*_ (m·s^−2^) and *c*_*spd*_ (s^−1^) and *b*_*E*_ (ml·kg^−1^·s^−2^) and *c*_*E*_ (s^−1^) represent the curvature constants describing the decrement of running speed (m·s^−1^) and *E* (ml·kg^−1^·s^−1^) respectively with increasing duration (*t* in seconds). *b*_*E*_*/c*_*E*_* and b*_*spd*_*/c*_*spd*_ were calculated using equations which were previously developed^32^.

The third hypothesis required determination of the agreement between experimental and predicted all-out running performances from the two trials equation based on RERI_E_ and RERI_spd_, as well as the validity of the two trials procedure to predict performances of world class runners utilizing any two run performances closely selected to world class run timings (Table 3).

All laboratory sessions were conducted at the Human Bioenergetics Laboratory in the Physical Education and Sports Science department of National Institute of Education, Singapore. Track tests were performed at the 400 m track located at the Sports and Recreation Centre at the Nanyang Technological University, Singapore. The laboratory tests were performed on a motorized treadmill (H–P Cosmos) with gradient set at 1% for all treadmill running protocols except for the maximal oxygen uptake ($\dot{\text V}{\text O_{2{\text {max}}}}$) protocol^33,34^. Athletes were strapped to an upper body safety harness to prevent them from falling. The harness did not assist or impede the athletes during the different tests.

### Experimental tests and measurements

#### Anthropometrical and body composition measurements

Prior to testing, height (Harpenden stadiometer, Holtain ltd, Britain) and weight (Metler Toledo GMBH, Germany) of the athletes were measured. A dual energy x-ray absorptiometry (DEXA) scan (DQR 4500W, Hologic Inc, Waltham, USA) scan was also performed to determine body composition^35^.

#### Astrand modified running continuous incremental maximal treadmill (AMRMAX) protocol

$\dot{\text V}{\text O_{2{\text {max}}}}$ was determined using the AMRMAX protocol. The test began with an initial speed of 8–12 km·h^−1^ with 0% gradient. After 3 min of running, gradient was increased by 2.5% at 2 min stages until volitional exhaustion. Capillary whole blood samples were taken from the finger at every minute for 5 min post exercise. BLa was analyzed via YSI 2300 STAT Plus (2300 D, YSI Incorporated, USA) to measure peak post exercise BLa. Breath-by-breath cardiorespiratory variables were analyzed and averaged at every 15 s. Heart rate (HR) was measured via a Polar HR transmitter (Polar Electro, Singapore) which sends its signals to the receiver of ParvoMedics TrueOne® 2400 metabolic system.

$\dot{\text V}{\text O_{2{\text {max}}}}$ was determined when athletes satisfied three of the following five criteria according to American College of Sports Medicine guidelines^36^; Applied Physiology of Exercise Chapter 5^37^; 1) Plateau of $\dot{\text V}{\text O_{2}}$ (change in $\dot{\text V}{\text O_{2}}$  ≤ 2.1 ml·kg^−1^·min^−1^ in spite of increasing treadmill gradient^38^), 2) Respiratory Exchange Ratio at $\dot{\text V}{\text O_{2{\text {max}}}}$ ≥ 1.1^39^, 3) BLa > 8 mmol·L^−1^^40,41^, 4) HR ≥ 90% of the age predicted maximal HR (HR_max_)^38^, and 5) volitional exhaustion.

#### Submaximal discontinuous treadmill (SUBMAX) protocol

A series of six to nine discontinuous submaximal treadmill runs were performed. Initial speed was set at approximately 40–60% $\dot{\text V}{\text O_{2{\text {max}}}}$, with 4–5% $\dot{\text V}{\text O_{2{\text {max}}}}$ increments at every stage depending on the ability of the athlete. All running speeds were within 40–90% $\dot{\text V}{\text O_{2{\text {max}}}}$^14^. Running sessions were fixed at 4 min, with 2–4 min recovery between sessions. Capillary blood samples were collected immediately after each submaximal running session through the finger prick technique. Steady state cardiorespiratory and aerobic metabolic measures were recorded at every 15 s during the 3^rd^ and 4^th^ minute of each treadmill running session.

$\dot{\text V}{\text O_{2}}$ and corresponding speeds were plotted, and a linear regression equation was obtained for each athlete^14^. This linear relation was extrapolated to the $\dot{\text V}{\text O_{2{\text {max}}}}$ attained during AMRMAX protocol and the corresponding speed was termed as velocity at $\dot{\text V}{\text O_{2{\text {max}}}}$ (v$\dot{\text V}{\text O_{2{\text {max}}}}$)^42–45^. Velocity at lactate threshold (vLT) was determined by plotting BLa values for each corresponding speed using the log–log plot method^46^, and velocity at delta 50 (υΔ50) calculated as the average of vLT and v$\dot{\text V}{\text O_{2{\text {max}}}}$.

#### $\dot{\text V}{\text O_{2}}$ till exhaustion (T_lim_) tests

T_lim_ tests were conducted at 100% v$\dot{\text V}{\text O_{2{\text {max}}}}$ (T_lim_v$\dot{\text V}{\text O_{2{\text {max}}}}$) and υΔ50. However, it was found that all but one participant was unable to reach $\dot{\text V}{\text O_{2{\text {max}}}}$ at υΔ50. Hence, 5%v$\dot{\text V}{\text O_{2{\text {max}}}}$ was added to υΔ50 for participants to achieve maximal aerobic energy during the T_lim_ test (υΔ50 ± 5%v$\dot{\text V}{\text O_{2{\text {max}}}}$). The speed at which maximal aerobic energy was attained during T_lim_ at υΔ50 and υΔ50 ± 5%v$\dot{\text V}{\text O_{2{\text {max}}}}$ was termed Vsub%95.

In each test, athletes started unassisted running at a pre-selected speed within 2–4 s and continued running until exhaustion. Time taken to exhaustion starting from unassisted running was recorded. Breath-by-breath cardiorespiratory measurements were also recorded in each run.

The breath-by-breath $\dot{\text V}{\text O_{2}}$ response recorded at T_lim_v$\dot{\text V}{\text O_{2{\text {max}}}}$ was interpolated per second and the time was aligned to the start of the run. The time was averaged at every 5 s via a moving average filter and the data was fitted to a positive exponential non-linear regression equation^47^ by means of weighted least square method using SigmaPlot software (windows version 11.0.0.77, Germany). The kinetics of $\dot{\text V}{\text O_{2}}$ at T_lim_v$\dot{\text V}{\text O_{2{\text {max}}}}$ was generally described by double or triple positive exponential regression equation.

Time to attain $\dot{\text V}{\text O_{2{\text {max}}}}$ (TA$\dot{\text V}{\text O_{2{\text {max}}}}$) during T_lim_v$\dot{\text V}{\text O_{2{\text {max}}}}$ and T_lim_Vsub%95 was calculated according to the primary criteria of achieving ≥ 95% $\dot{\text V}{\text O_{2{\text {max}}}}$^47^. If the athletes did not achieve the primary criteria of TA$\dot{\text V}{\text O_{2{\text {max}}}}$ at T_lim_v$\dot{\text V}{\text O_{2{\text {max}}}}$, the secondary criteria to determine TA$\dot{\text V}{\text O_{2{\text {max}}}}$ were employed^32^. Subsequently, time at maximal aerobic energy (${\text{T}}_{\text{lim}}\dot{\text V}{\text O_{2{\text {maxconverted}}}}$) and T_lim_$\dot{\text V}{\text O_{2{\text {maxconverted}}}}$ during T_lim_v$\dot{\text V}{\text O_{2{\text {max}}}}$ were computed using Eqs. 3^47^ and 4.

3 $$T_{\lim } \dot{V}{O_{2 \text{max}}} v\dot{V}{O_{2{\text {max}}}} = T_{\lim } v\dot{V}{O_{2 \text{max}}} - TA\dot{V}O_{2 \text{max}} v\dot{V}O_{2 \text {max}}$$

4 $$T_{\lim } \dot{V}O_{2 \text {maxconverted}} \left( s \right) = \frac{{(T_{\lim } \dot{V}O_{2 \text {max}} v\dot{V}O_{2 \text {max}} \times v\dot{V}O_{2 \text {max}} )}}{Vsub\% 95 }$$

#### Speed and duration curve protocol

A minimum of two to three trials were performed at different speeds ranged between 90%v$\dot{\text V}{\text O_{2{\text {max}}}}$ and 140%v$\dot{\text V}{\text O_{2{\text {max}}}}$. The duration up to exhaustion at each run speed was recorded. Participants were given sufficient recovery between the trials and were allowed to discontinue the test if they were unable to sustain their best effort.

The hyperbolic relation (Eq. 5) was fitted to speed data and corresponding duration calculated during the different T_lim_ sessions as well as the speed and duration curve protocol. Speeds in the range of 90%v$\dot{\text V}{\text O_{2{\text {max}}}}$ to 140%v$\dot{\text V}{\text O_{2{\text {max}}}}$ were considered and the anaerobic distance capacity (ADC) and critical speed (CS) were determined for each participant.

5 $$MAS \left( {m\cdot s^{ - 1} } \right) = CS + \left[ {\frac{ADC}{{B + MAS_{dur} }}} \right]$$

where, CS represents critical speed, ADC represents anaerobic distance capacity, MAS_dur_ represents duration of MAS, and B represents constant.

#### Determination of maximal aerobic speed (MAS)

MAS is defined as the minimum speed at which maximal aerobic energy is elicited with negligible contribution from anaerobic energy sources during a sustained run. MAS_dur_ was calculated using the equation MAS_dur_ = T_lim_Vsub%95 − (– ${\text{T}}_{\text{lim}}\dot{\text V}{\text O_{2{\text {maxconverted}}}}$) (Eq. 6). MAS was then determined using the hyperbolic relation between speed and duration for each athlete. This MAS was determined using a backwards validation by reducing the error of prediction by making the concept of MAS determination accurate. This accuracy of MAS is indicated by the low approximate prediction error of 0.2–2.7% as shown in Table 3. The accuracy of this validation has been shown in Part 1 of this study^31^.

#### 50 m sprint run, 200 m, 400 m test

The 50 m sprint run was performed with a standing start position at the start line. At the start command, the athlete accelerated and covered the distance of 50 m in the least possible time. The speed and time at the stipulated distance intervals within 50 m were automatically recorded by the five timing gates placed within 34–50 m for sprinters and middle distance runners and within 30–46 m for endurance athletes. A minimum of two trials were performed, with a 15–20 min rest interval in between the trials, and the best performance was recorded to the nearest 0.01 s. Both 200 m and 400 m speed trials were conducted on different days according to the 50 m sprint run test procedures. The time taken for each race was recorded to the nearest 0.01 s. The measured time and speeds at specific intervals during 50 m sprint run test were analyzed and the highest speed among all recorded speeds was determined as maximal anaerobic speed (MAnS).

#### 1500 m, 3000 m, 5000 m test

Participants ran each distance on different days at their own self-regulated pace. They were briefed to run at their targeted best effort based on their fitness level. The time taken to cover each run was recorded to the nearest 0.01 s.

Breath-by-breath aerobic variables were measured via a portable metabolic analyzer (ParvoMedics TrueOne® 2400, ParvoMedics, Inc, USA). $\dot{\text V}{\text O_{2{\text {max}}}}$ and carbon dioxide (CO_2_) sensors were calibrated for daily variations in barometric pressure, temperature and the humidity of the testing environment. Flow meter was calibrated via a 3.000L volume calibration syringe (Hans Rudolph, MO, USA), and HR was measured via a Polar HR transmitter (Polar Electro, Singapore). Automated BLa analyzer (YSI 2300 STAT Plus, 2300D, YSI Incorporated, USA) was utilized for analyzing the BLa samples.

#### E_MAS_ and E_MAnS_, and Mapping of RERI_E_ and RERI_spd_

The linear relation between speed and $\dot{\text V}{\text O_{2{\text {max}}}}$ measured through the SUBMAX protocol was extrapolated to MAS and MAnS. The amount of extrapolated energy at these maximal speeds were considered as maximal aerobic (E_MAS_) and maximal anaerobic energy (E_MAnS_) respectively ^5^.

RERI_E_ and RERI_spd_ were then computed via Eqs. (7) and (8).

7 $$RERI_{E} = \frac{{E_{{{\text{MAnS}}}} \left( {{\text{ml}} \cdot {\text{kg}}^{ - 1} \cdot {\text{s}}^{ - 1} } \right) }}{{E_{{{\text{MAS}}}} \left( {{\text{ml}} \cdot {\text{kg}}^{ - 1} \cdot {\text{s}}^{ - 1} } \right)}}$$

8 $$RERI_{spd} { } = \frac{{{\text{MAnS }}\left( {m \cdot s^{ - 1} } \right){ }}}{{{\text{MAS }}\left( {m \cdot s^{ - 1} } \right)}}$$

### Statistical analysis

The number of running trials for an athlete was indicated as *n*_*T*_. Paired *t* tests were employed to determine the significant difference between RERI_E_ and RERI_spd_ and coefficient of determination was calculated to determine their relationship (hypothesis 1). Multiple regressions were performed with the data to significantly account for the prediction of performance in 200 m, 5000 m track runs and other treadmill running trials (hypothesis 2). The difference between the value of *c*_*spd*_ and *c*_*E*_ among ST, MD, and ET athletes was determined with the analysis of variance (ANOVA) statistical technique (hypothesis 2). The goodness of fit (*R*^*2*^) and percent of error of RERI model (hypothesis 2) and two trials equation (hypothesis 3) in predicting running performances were calculated. Level of significance was set at *p* ≤ 0.05.

## Results

### Hypothesis 1: RERI_E_ and RERI_spd_ between athletes

**Figure 1:**
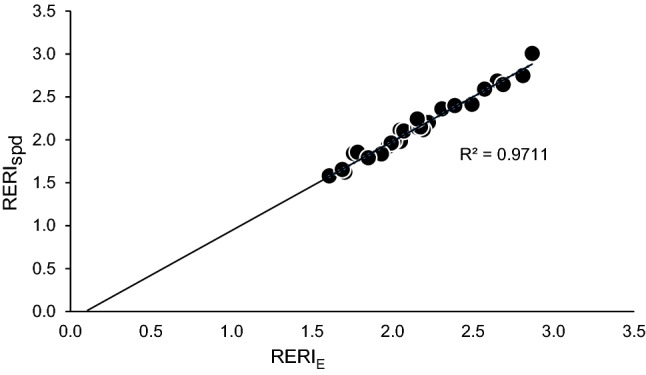
Relation between RERI_E_ and RERI_spd_.

Paired *t*-tests indicated no significant difference (*p* = 0.28) between RERI_E_ (2.15 ± 0.34) and RERI_spd_ (2.13 ± 0.36) among all athletes. High correlation was found between RERI_E_ and RERI_spd_ (r = 0.99, *p* < 0.001), with a high agreement (R^2^ = 0.97) (Fig. 1).

### Hypothesis 2: value of constant c

The average values of *c*_*E*_ and *c*_*spd*_ were found to be at 0.0185 ± 0.003 s^−1^ and 0.0185 ± 0.003 s^−1^ respectively. As there was no significant difference found between both constants in all runners (*p* = 0.678), these constants are indicated as c for both models based on RERI_E_ and RERI_spd_ and was fixed at 0.0185.

### Hypothesis 2: prediction of performances via model based on RERI_E_ and RERI_sp_

**Figure 2:**
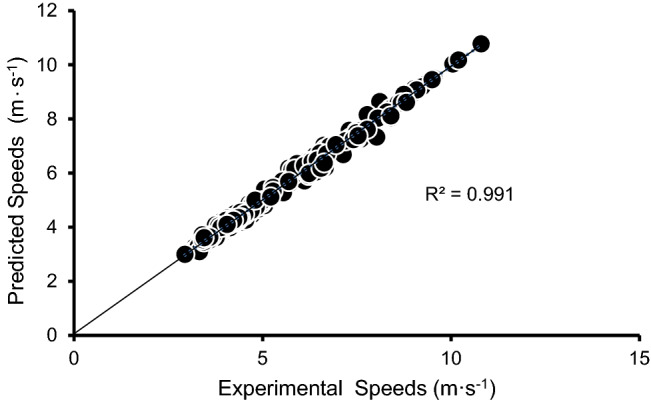
Experimental and predicted speeds on track and treadmill utilizing RERI_E_ model.

RERI_E_ and RERI_spd_ significantly differentiated between ST, MD, and ET athletes (Table 1). The RERI_E_ model with a fixed *c*_*E*_ value of 0.0185 s^−1^ predicted the all-out running performances to within an average of 2.39 ± 2.04% (*R*^*2*^ = 0.99, *n*_*T*_ = 252) for all athletes (Fig. 2), with treadmill trials to within an average of 2.26 ± 1.89% (*R*^*2*^ = 0.99, *n*_*T*_ = 203) and track trials to within an average of 2.95 ± 2.51% (*R*^*2*^ = 0.99, *n*_*T*_ = 49).

**Table 1:** Descriptive variables of athletes.

Variable	ST	MD	ET	Total
N	6	6	6	18
MAS (m·s^−1^)	3.64 ± 0.17^†^	4.13 ± 0.22^¥^	4.70 ± 0.44**	4.16 ± 0.53^§§^
MAnS (m·s^−1^)	9.86 ± 0.61^†^	9.07 ± 0.19^¥^	8.17 ± 0.38**	9.03 ± 0.82^§§^
E_MAS_ (ml·kg^−1^·s^−1^)	0.78 ± 0.06	0.88 ± 0.06	0.93 ± 0.08*	0.86 ± 0.09^§^
E_MAnS_ (ml·kg^−1^·s^−1^)	2.05 ± 0.12	1.99 ± 0.09^¥¥^	1.68 ± 0.14*	1.91 ± 0.20^§§^
RERI_spd_	2.71 ± 0.11^††^	2.20 ± 0.11^¥¥¥^	1.75 ± 0.14**	2.22 ± 0.42^§§^
RERI_E_	2.63 ± 0.12^††^	2.27 ± 0.13^¥¥¥^	1.82 ± 0.15*	2.24 ± 0.36^§§^
Measured 200 m speed (m·s^−1^)	8.11 ± 0.54^†^	7.50 ± 0.20	6.94 ± 0.35**	7.52 ± 0.61^§§^
Measured 5000 m speed (m·s^−1^)	3.46 ± 0.14	3.90 ± 0.22	4.37 ± 0.61*	3.91 ± 0.53^§^

Values are in means ± SD. N: number of trials; MAS: maximal aerobic speed; MAnS: maximal anaerobic speed; E_MAnS_: oxygen uptake at MAnS or maximal anaerobic energy; E_MAS_: oxygen uptake at MAS or maximal aerobic energy; RERI_E_: running energy reserve index based on maximal energies, E_MAnS_ and E_MAS_; RERI_spd_: running energy reserve index based on maximal speeds, MAnS and MAS. ST: sprint-trained; MD: middle distance; ET: endurance trained. Significant difference between ET and ST, *p < 0.01, **p < 0.001*; Significant difference between ST and MD, *^*†*^*p* < 0.05*, *^*††*^*p* < 0.001*; Significant difference between ET and MD, *^*¥*^*p* < 0.05*; *^*¥¥*^*p* < 0.01*; *^*¥¥¥*^*p* < 0.001*; Significant difference between all groups, *^*§*^*p* < 0.01*, *^*§§*^*p* < 0.001*.*

### Hypothesis 3: prediction of all-out running performances based on two trials equation

Three combinations of durations and distances based on the two trials procedure were utilized to predict performances.

#### 3000 m & 50 m, 745 s & 4 s, 3000 m & last 13 m of 50 m sprint

**Figure 3:**
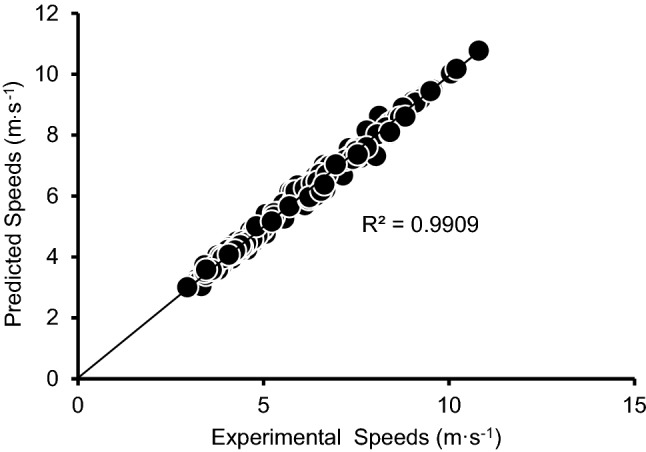
Experimental and predicted speeds on track and treadmill utilizing RERI_spd_ model.

The algorithm based on two track performances in Figs. 2 and 3 were comparable to those predicted from 3000 m & 50 m, to 745 s & 4 s (Table 2). 3000 m and speed of last 13 m ~ during 50 m sprint test were used in the two trials equation to predict running performances of three participants in various distances. This procedure predicted all-out track performances within errors of 2.43% (Table 2).

**Table 2:** Predictive accuracy of two trials equation using different set of durations and distances.

Combinations of distance and durations	Type of two running trials	Prediction trials	n_T_	R^2^	Error of RERI_E_ (%)	Error of RERI_spd_ (%)
3000 m and MAnS	Treadmill & track	Treadmill & track	216	0.97	2.06	2.02
5000 m and MAnS	Track	Treadmill & track	46	0.97	2.22	1.93
745 and 4 s ~	Treadmill	Treadmill and track	193	0.97	2.56	2.59
3000 m and last 13 m ~ of 50 m sprint	Track	Track	12	0.99	2.43	2.43

n_T_, number of trials; R^2^: Goodness of fit of two trials equation; RERI_E_: ratio of $\dot{\text V}{\text O_{2{\text {max}}}}$ at maximal anaerobic speed to $\dot{\text V}{\text O_{2{\text {max}}}}$ at maximal aerobic speed; RERI_spd_: ratio of maximal anaerobic speed to maximal aerobic speed.

In addition, the two trials equation based on RERI and constant c fixed at 0.0185 also predicted world class running performances of athletes such as Haile Gebrselassie, Sebastian Coe, Kenenisa Bekele, EI Guerrouj and other world class athletes (Table 3).

**Table 3:** Prediction of world class performances using two trials equation based on RERI_spd_ model.

Name	Country	Gender	Year	Two trials	Predicted performances in distances in m (time in s)	Spd_act_ (m·s^−1^)	Spd_pred_ (m·s^−1^)	Spd_pre_/Spd_Act_ ratio	% error
K. Bekele	ETH	M	2003	3000 & 1500	3218 (493.51)	6.5	6.7	1.03	2.7
K. Bekele	ETH	M	2003	3000 & 1500	5000 (769.53)	6.5	6.6	1.01	1.3
Cram S	GBR	M	1985	800 & 2000	1000 (132.88)	7.5	7.5	1.00	0.2
Cram S	GBR	M	1985	800 & 2000	1500 (209.67)	7.2	7.1	0.99	1.00
Cram S	GBR	M	1985	800 & 2000	1609 (226.32)	7.1	7.0	0.99	1.3
Cruz Joaquim	BRA	M	1984	1000 & 1609	800 (101.77)	7.9	7.8	0.99	0.9
de Souza H	BRA	M	2006	1000 & 3000	800 (107.85)	7.4	7.4	1.00	0.1
de Souza H	BRA	M	2006	1000 & 3000	5000 (822.56)	6.1	5.9	0.98	2.4
H. Gebrselassie	ETH	M	1998	1500 & 3000	5000 (759.36)	6.6	6.7	1.01	1.4
EI G. Hicham	MAR	M	2003	1500 & 3000	1609 (230.2)	7.0	7.1	1.02	1.8
EI G. Hicham	MAR	M	2003	1500 & 3000	5000 (770.24)	6.5	6.4	0.99	0.8
Kamel Yusuf S	BRN	M	2008	1000 & 1500	800 (102.79)	7.8	7.7	0.99	1.3
Komen D	KEN	M	1995	1500 & 3000	5000 (776.15)	6.4	6.4	0.99	1.2
Masterkova S	RUS	F	1996	1000 & 1609	800 (116.04)	6.9	6.9	1.00	0.2
Masterkova S	RUS	F	1996	1000 & 1609	1500 (236.77)	6.3	6.4	1.01	1.1
Mottram Craig	AUS	M	2001	1500 & 3000	1609 (233.06)	6.9	6.9	1.00	0.0
Mottram Craig	AUS	M	2001	1500 & 3000	2000 (306.97)	6.5	6.7	1.03	3.1
Mottram Craig	AUS	M	2001	1500 & 3000	5000 (803.94)	6.2	6.3	1.01	1.3
Ngeny Noah	KEN	M	2000	1000 & 3000	800 (104.49)	7.7	7.7	1.00	0.2
Ngeny Noah	KEN	M	2000	1000 & 3000	1500 (208.12)	7.2	7.1	0.98	2.2
Ngeny Noah	KEN	M	2000	1000 & 3000	1609 (227.67)	7.1	7.0	0.99	1.2
O'Sullivan S	IRL	F	1993	1000 & 3000	1500 (239.6)	6.3	6.2	0.99	0.9
O'Sullivan S	IRL	F	1993	1000 & 3000	1609 (262.94)	6.1	6.2	1.01	0.6
O'Sullivan S	IRL	F	1993	1000 & 3000	5000 (885.92)	5.6	5.8	1.02	2.1
Sebastian Coe	GBR	M	1981	200 & 1000	400 (47.05)	8.5	8.3	0.98	2.1
Sebastian Coe	GBR	M	1981	200 & 1,000	800 (101.7)	7.9	7.7	0.98	1.5
Sebastian Coe	GBR	M	1981	200 & 1000	1609 (227.33)	7.1	7.3	1.03	2.5
Spivey Jim	USA	M	1995	1500 & 3000	2000 (299.19)	6.7	6.6	0.99	1.3
Mohamed, S	QTR	M	1995	1500 & 3000	1609 (231.12)	7.0	6.9	1.00	0.3
Mohamed S	QTR	M	1995	1500 & 3000	2000 (295.57)	6.8	6.7	0.99	1.0
G. Markos	ETH	M	2005	1500 & 3000	5000 (780.25)	6.4	6.4	0.99	0.9

Source of data: International Association of Athletics Federations (IAAF); %error: error in prediction of running performance; Spd_pred_/Spd_act_, ratio of predicted and actual speed. Table adapted from Gupta^32^.

## Discussion

### Comparison of RERI_E_ and RERI_spd_ model and prediction of track and treadmill running performances

This study found no significant difference between the RERI_E_ and RERI_spd_ of athletes, confirming the first hypothesis. These results seemed valid as RERI_E_ and RERI_spd_ measurements were based on the linear relationship between $\dot{\text V}{\text O_{2{\text {max}}}}$ and run speeds, with RERI_E_ and RERI_spd_ representing the X and Y-axis respectively in Fig. 1. Significant correlations were found between MAS and E_MAS_ (r = 0.819, *p* < 0.001) and between MAnS and E_MAnS_ (r = 0.737, *p* < 0.001). This indicated a relation between maximal energies and maximal speeds as well as between RERI_E_ and RERI_spd_.

The main finding of this study was that the RERI_E_ and RERI_spd_ model based on rational mathematical functions accurately predicted approximately 5–1340 s treadmill, 200 m track and 5000 m track all-out running performances. While Bundle’s AnSR procedure was able to predict track and treadmill running performances to within an average of 3.4% (*R*^*2*^ = 0.86, *n*_*T*_ = 28) and 2.5% (*R*^*2*^ = 0.94, *n*_*T*_ = 84) respectively, it was only able to predict all-out running performances up to 240 s. A model by di Prampero et al. ^22^ was able to predict running performances beyond 240 s. This model was based on measurement of energy cost of running, $\dot{\text V}{\text O_{2{\text {max}}}}$, and hypothetical values of AnS (maximal amount of anaerobic energy release), and also predicted 800–5000 m middle distance run performances. However, the accuracy was lower than that of the RERI model (di Prampero’s model: 800 m = 1.16 ± 0.09, 13.8%; 1000 m = 1.07 ± 0.09, 7.5%; 1500 m = 1.04 ± 0.06, 5.9%; 3000 m = 1.03 ± 0.066, 5.3%; 5000 m = 1.03 ± 0.02, 2.4%^22^; RERI model: 800 m ~  = 0.97 ± 0.02, 2.9%; 1500 m ~  = 1.00 ± 0.03, 2.2%; 3000 m ~  = 1.01 ± 0.02, 1.3%; 5000 m ~  = 0.99 ± 0.03, 2.3%).

Many theoretical models are able to predict world-class performances with a 2% or less accuracy^21^. For example, the model developed by Peronnet and Thebault^21^ predicted performances of 60 m to marathon distance within an average of 0.73%. However, the validity of this model, as well as other theoretical models based on hypothetical or theoretical values of various parameters for predicting athletic performances in reality remain unknown^48^. In this study, all parameters were measured and only one fixed constant (c) was used after validating its value with actual experimental data.

In addition, rather than using exponential or hyperbolic mathematical functions, this study utilized the mathematical expression based on first-degree polynomial or rational nonlinear function. This expression best fitted with the data of estimated energy at corresponding durations. E_MAnS_ and MAnS theoretically define the point of estimated energy demand and speed, respectively, at which duration equals to zero. Since time at E_MAnS_ and MAnS was less than a second, using E_MAnS_ and MAnS as parameters of these models are well defined as the upper most measurable limit of maximal energy and speed of the athletes. Although the validity of extrapolating the maximal anaerobic energy at MAnS has not been established, it has predicted running performances with high accuracy in previous studies^5,48^ as well as in the present study. b_E_/*c*_*E*_ and *b*_*spd*_/*c*_*spd*_ are theoretically defined as energy demand and speed corresponding to each other, at which the athlete may be able to run with the utilization of maximal aerobic energy for a longer time. *b*_*E*_ and *b*_*spd*_ are directly related to E_MAS_ and MAS respectively, and c was experimentally fixed at 0.0185 with no significant difference among athletes. Similar results were shown when c was measured with the data of speeds and corresponding run durations.

When applied along with determining MAS and MAnS parameters and corresponding energies on a wide variety of data, all-out running speeds of duration ranging between approximately 5–1500 s were accurately predicted. These results confirmed the second hypothesis and indicate that the RERI model derived from the data of metabolic energy and run speeds as a function of run duration is applicable and can accurately predict all-out run performances.

### Prediction of all-out track and treadmill running performances using two-trial equation based on RERI_E_ and RERI_spd_ model

The RERI procedure with two trials equation was developed as an alternative to the time consuming procedures of measuring E_MAS_, E_MAnS_, MAS, MAnS, RERI_E_, and RERI_spd_. The two trials equation was based on RERI_E_ and predicted all-out running trials with accuracy comparable to that of the long derived method to predict performance. However, this procedure required the measurement of energy demand at different speeds using the metabolic system. The two trials equation based on RERI_spd_ was standardized such that it was similar to the model based on RERI_E_. This two trials equation using 3000 m on treadmill and 50 m sprint on track, 745 s and 4 s on treadmill, and 3000 m and 13 m track speeds predicted running performances with similar accuracy to that based on RERI_E_ model. In addition, RERI_spd_ was also able to predict all-out running performances in the absence of a metabolic measurement system.

The effect of a slight change in duration or distance of MAS in the two trials equation may have an effect on the accuracy of determining RERI and predicting all-out running trials. However, the two trials equation based on the RERI_E_ and RERI_spd_ models predicted all-out running performance of treadmill and track with similar accuracy to those predicted from other combinations of two trials procedure. This suggest that small variations in distances to predict the remaining all-out performances may not significantly affect its accuracy.

The accuracy of the two trials equation is comparable to Bundle et al.^5^ two trials prediction equation, which was based on all-out performances of 3 s and 60 s run on the treadmill and a peak speed in 55 m sprint and 400 m all-out run performance on track. Bundle and colleagues predicted the 3–240 s treadmill performances to within an average of 3.7% (*R*^*2*^ = 0.93, *n*_*T*_ = 77) and 100–400 m track performances to within an average of 3.3% (*R*^*2*^ = 0.89, *n*_*T*_ = 28). Using peak speed in the 55 m and 400 m speed on track, Bundle et al.^11^ predicted 100–400 m track and 3–240 s treadmill running performances to within average of 3.1% (*R*^*2*^ = 0.91, *n*_*T*_ = 21) and 4.1% (*R*^*2*^ = 0.86, *n*_*T*_ = 84) respectively. However, Bundle’s procedure only predicted all-out run performances up to 240 s. The RERI model based on Eqs. (1) and (2), as well as the two trials equation accurately predicted running performances up to 5000 m. The RERI model also reduces the use of sophisticated equipment by utilizing the last 13 m of the 50 m sprint run instead of MAnS to predict all-out run speeds. This was calculated using various statistical procedures to determine the two best suited distances which could determine speeds close to MAS and MAnS and their corresponding oxygen consumptions. The accuracies of the two trials equation using speeds of 4 s and 745 s treadmill all-out runs were 2.56% (RERI_E_) and 2.59% (RERI_spd_). 3000 m track performances, along with the last 13 m during the 50 m sprint run predicted track performances of 200 m, 400 m, 1500 m, and 5000 m of the three participants to within an average of 2.43% (*R*^*2*^ = 0.99, *n*_*T*_ = 12), which was higher than Bundle’s AnSR method (4.1%, *R*^*2*^ = 0.86, *n*_*T*_ = 84). Thus, the RERI model may be more suitable and accurate compared to Bundle et al.’s AnSR in predicting all-out running performances. The two trials equation may be a promising alternative for predicting running performances and significantly categorizing athletes accurately with ease of administration.

The two trials procedure predicted running performances with high accuracy of 1.25% (*R*^*2*^ = 0.98, *n*_*T*_ = 31) and the ratio of predicted and actual performances was 1.00 ± 0.02 (Table 3) for world class performances. Specifically, the two trials equation predicted short and middle performances to within an average of 2.1% (400 m, N = 1), 0.7% (800 m, N = 6), 1.1% (1000–1609 m, N = 12), 2.0% (2,000–3,218 m, N = 4), and 1.4% (5000 m, N = 8) as shown in Table 3. Additionally, to compare the accuracy of the RERI model with Bundel’s AnSR, the 800 m and 1000 m performance of Sebastian Coe were predicted using his world class performances of 400 m and 1609 m in 1981. The predictive accuracy was 1.8% and 1.0% for his 800 m and 1000 m respectively which was comparable to that of Bundle’s two trials equation (1000 m = 1.5% and 800 m = 2.0%). These results suggest that accurate prediction of the running performances of world class runners is possible when utilizing any two running performances within 5000 m distance performance. The results also indicate that the RERI model may prove to be an accurate alternative to time consuming methods of determining anaerobic and aerobic performances^49,50^, and anaerobic energy^14^.

## Conclusion

This study concluded that the RERI_E_ model may be able to predict track performances of 200 m and 5000 m, and treadmill performances ranging between 5 and 1340 s with a high level of accuracy. The RERI_spd_ model, which was validated against RERI_E_, also predicted all-out running trials with a high level of accuracy. The brief RERI procedure based on two trials equation predicted all-out running performances with a high precision similar to long derived model. Using the last 13 m during 50 m sprint run test and 4 s run speed on treadmill along with 3000 m and 745 s respectively proves to be accurate and convenient in predicting all-out running performances. The RERI model is a non-invasive and convenient method that may be used to differentiate between different categories of athletes, as well as identify future athletes based on their energy. In addition, the two trials procedure can be used to determine short and middle distance running performances of athletes and world class runners. It may address some of the limitations of the current anaerobic techniques and mathematical models by determining the anaerobic energy reserve of athletes, accurately predicting athletic potentials and all-out running performances. The RERI model is applicable in predicting all-out running performances of athletes engaged in sprint to endurance training.

## Data Availability

All data generated or analysed during this study are included in this published article.
